# Account of Diseases in the Finsbury Dispensary, from March 20, to April 20, 1805

**Published:** 1805-05-01

**Authors:** J. Reid

**Affiliations:** Grenville-street, Brunswick-square


*Account of Diseases in the Finsbury Dispensary, from March 20, to April 20,* 1805.

| | | | |
|---|---|---|---|
| Rheumatismus - - - | 9 | Chlorosis - - - - - | 15 |
| Pneumonia - - - - | 6 | Icterus - - - - - - - | 3 |
| Hæmoptysis - - - - | 3 | Dysenteria - - - - - | 11 |
| Phthisis - - - - - | 12 | Morbi Cutanei - - - | 13 |
| Menorrhagia - - - - | 7 | Morbi Infantiles - - - | 16 |
| Leucorrhœa - - - - | 7 | Asthenia - - - - - - | 19 |
| Apoplexia - - - - - | 2 | Neuroses - - - - - | 16 |
| Paralysis - - - - - - | 8 | | |

The Reporter has lately been called to several cases of what were simple asthenia, but which had been previously regarded, and treated as inflammatory affections of either the thoracic or abdominal viscera.

Pain is generally, but in many instances unjustly, considered as an evidence of inflammation. It more frequently arises from the difficulty with which a debilitated and obstructed organ performs its accustomed and salutary office. Blisters relieve pain; but it is not so much by their evacuating as their stimulating operation. They are cutaneous drains. In cases, however, of extreme or chronic debility, such external applications are calculated to aggravate the degree, and perpetuate the existence of that weakness with which the patient is already afflicted. By acting upon the nervous system, or the sensorial power, blisters cannot be of any permanent or essential service. What produces pain cannot relieve pain, except by diverting the attention from one uneasy feeling to another. But, in such instances, the influence of the imagination is vague, and almost unlimited in its extent. The remedy prescribed has not so much effect as the reputed character of the prescriber. A patient who feels an imperfect faith in his physician, is not likely to experience the due efficacy of his medical direction. And, on the other hand, an entire confidence in the information and talents of a professional adviser, not seldom gives a salutary efficacy to remedies which would, otherwise, have been insufficient, or injurious in their physical operation.

Patients have recently occurred, in the practice of the Reporter, among both the elegant and vulgar classes of society, who were affected with what are called nervous diseases; a class, in nosology, which may be made to comprehend, or to be connected with, nearly all the corporeal as well as mental afflictions to which the human frame frame is liable. Nervous diseases are, in general, treated with too little delicacy and respect. No maladies are calculated more to interest the feelings of a humane man, or are more likely to receive alleviation, if not a radical cure, from a skilful and attentive practitioner. But, in such cases, the operation of moral agents ought to be more especially studied and applied.

The feelings of the mind should be watched and examined more than the functions of the body. Medicine, in such instances, is of important advantage; but amusement, or more serious occupation, is of radical and essential consequence.

In several instances, which the writer of this article has recently had an opportunity of observing, opium has not produced the intended and desired state of sleep and composure, but, on the contrary, has given rise to restlessness, and symptoms of morbid irritability and delirium. On which account he had recourse to hyoscyamus* as a substitute; which, while it acts as an opiate, is not followed, in its operation, by those inconvenient and obnoxious consequences which are apt to arise from the administration of laudanum, or of the drug in its solid form.

There is an extreme difficulty, more particularly in nervous diseases, in adjusting the dose, and selecting the particular formula of pharmaceutical prescription, as its efficacy depends so much on the uncertain susceptibility of the patient, which, in some instances, is extended to the highest, and, in others, reduced to the lowest degree of irritability, and capacity of excitation. "On comparing the situation of an hysterical female (says a modern author of ingenuity and eloquence) liable to distressing agitations from the most trifling causes, as the dropping of a hair-pin on the floor, to that of the engineer who stands unmoved amid the thunder of a battery; of the seaman who maintains his footing on the deck or ropes of his vessel, reeling under the shock of the elements; or of the Indian who exhibits the signs, and probably feels the throb of intense delight, while the flames are preying upon his flesh: how astonishing do we find the range in human susceptibility to the effect of the powers by which we are surrounded!"

J. REID.

*Grenville-street, Brunswick-square,* *April* 25, 1805.

---

* Hyoscyamus was a favourite remedy with Dr. Rutherford, of Edinburgh, for whose character and skill the writer of this article shall always feel the highest degree of reverence and esteem.

